# Supplementary material for: Tissue-Specific Microparticles Improve Organoid Microenvironment for Efficient Maturation of Pluripotent Stem-Cell-Derived Hepatocytes
Source: Cells. 2021 May 21;10(6):1274. doi: 10.3390/cells10061274 (PMC8224093; doi:10.3390/cells10061274)
Supplement: Supplementary file 1 [file cells-10-01274-s001.zip › Cells-1163560-Supplementary update 0527.pdf]

cells

MDPI

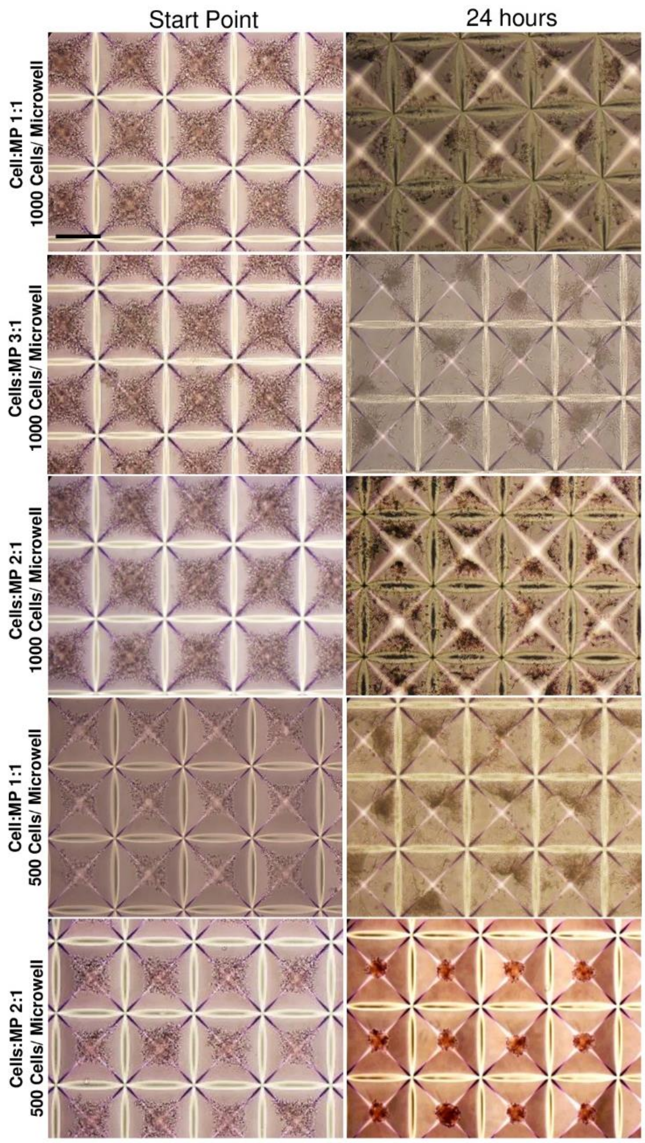

Figure S1. Optimization of cell/MP and cell/microwell ratio for successful cell aggregate formation. The cell combination consisted of HE:HUVEC:MSC (10:7:2). (MPs: microparticles; HE: hepatic endoderm; HUVEC: human umbilical vein endothelial cells; MSC: mesenchymal stem cell).

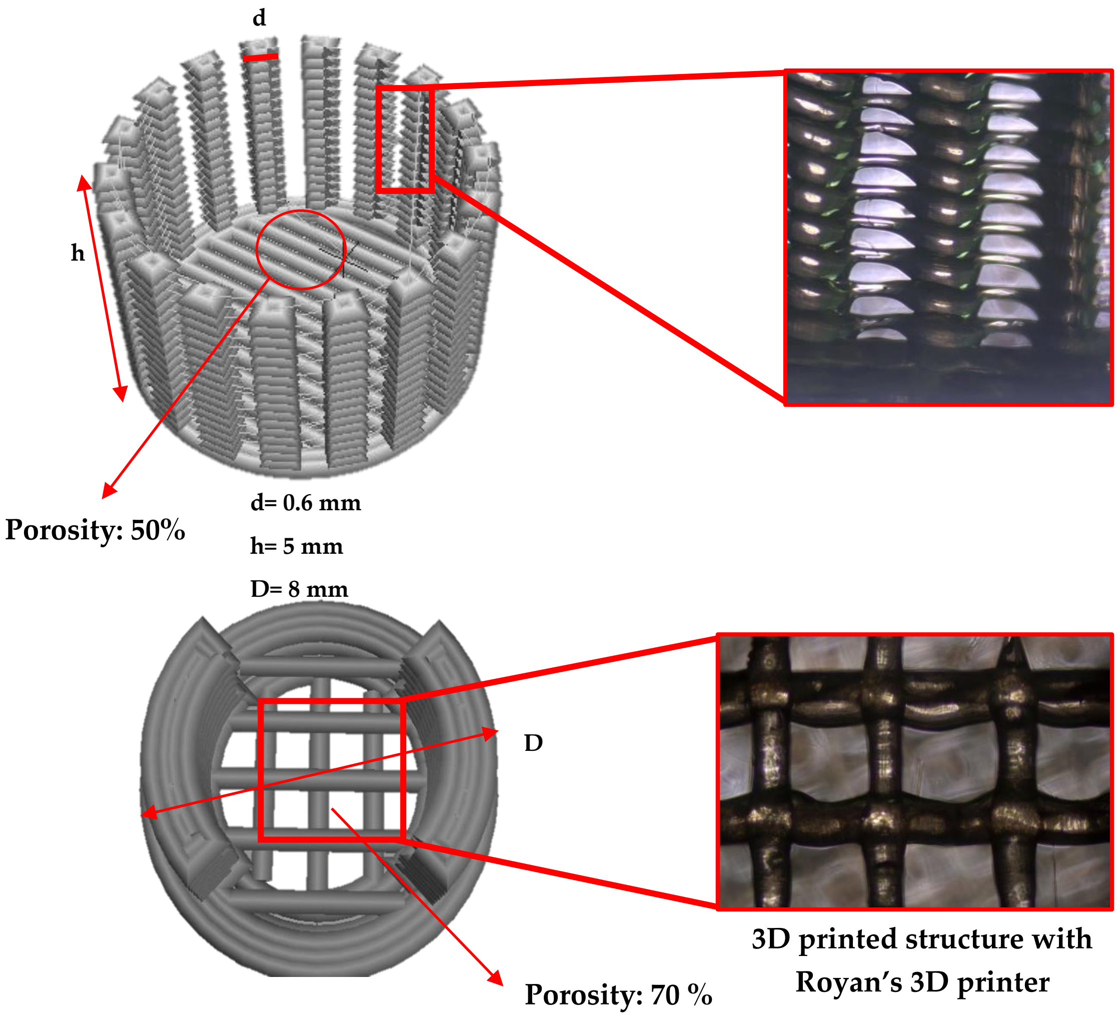

Figure S2. Fabrication of 3D basket by 3D printing. Schematic images of the basket and the microspcopy images of the printed structures.

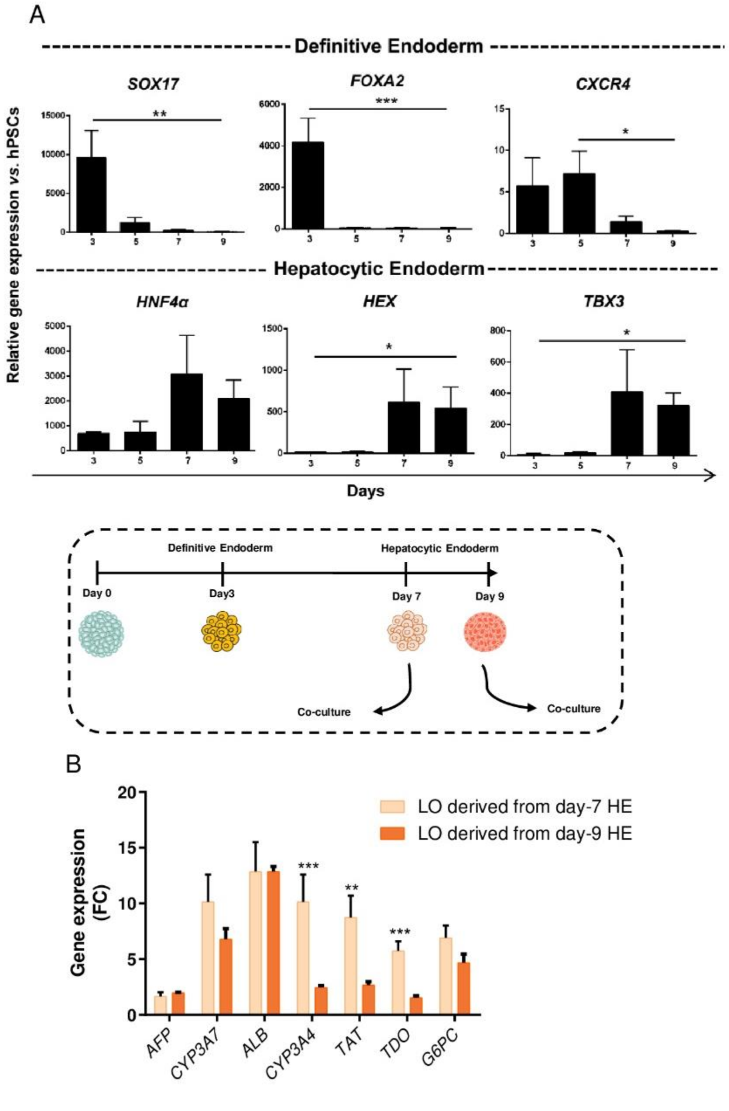

Figure S3. Determination of optimal conditions for differentiation of human embryonic stem cells into hepatic endoderm by gene expression analysis. **(A)** Relative gene expression analysis of definitive endoderm markers (*SOX17*, *FOXA2*, and *CXCR4*) and hepatic endoderm markers (*HNF4α*, *HEX*, and *TBX3*) on days 3, 5, 7, and 9 of differentiation. **(B)** Relative gene expression of mature hepatic markers (*ALB*, *CYP3A4*, *TAT*, *TDO*, and *G6PC*) and immature hepatic markers (*AFP* and *CYP3A7*) in liver organoids derived from day-7 and -9 HE. Data are shown as mean±SD (n = 3). Statistical analysis was performed by using unpaired two-tailed Student's t-test. * $p < 0.05$, ** $p < 0.01$, *** $p < 0.001$. (HE: hepatic endoderm).

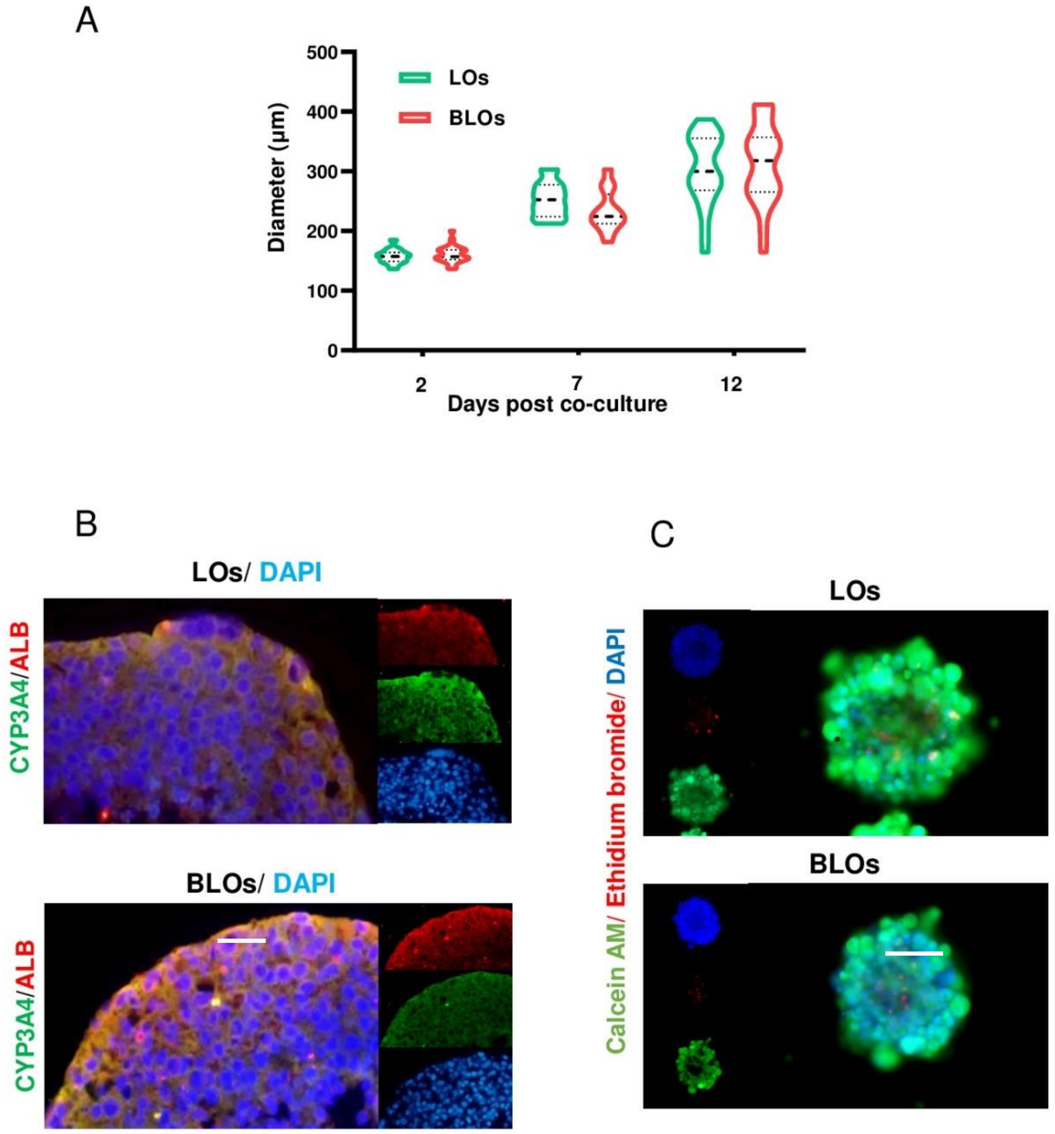

Figure S4. Size distribution of the cell aggregates on different days post-co-culture, and immunostaining and live and dead staining of LOs and BLOs (**A**)Size distribution of the cell aggregates on different days post-co-culture. (**B**) Immunostaining of BLOs and LOs derived from hESCs for ALB, CYP3A4, CYP1A2. The nuclei were counterstained with DAPI (Scale bar:100 μm). (**C**) Live and dead staining of BLOs and LOs derived hESCs (Scale bar: 200 μm).

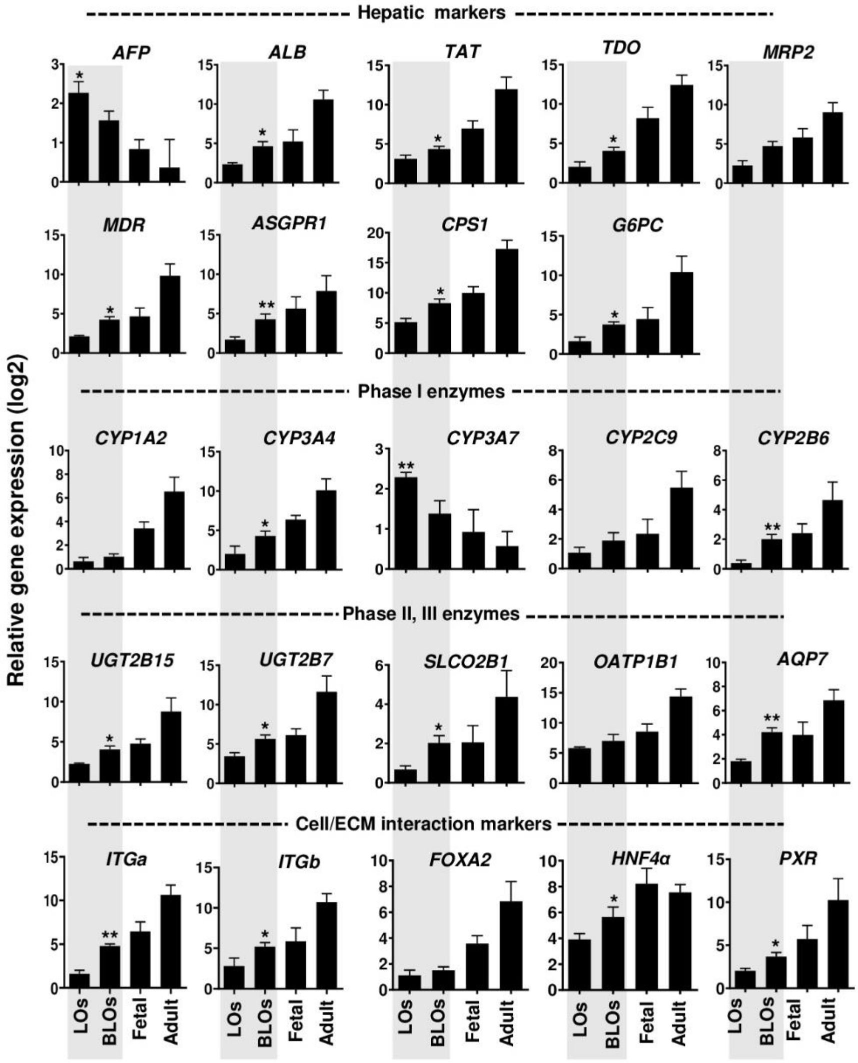

Figure S5. Gene expression profile in the LOs and BLOs derived from 7-day HE differentiated from induced pluripotent stem cells (iPSC4 cell line). Relative gene expressions of hepatic-specific genes (*AFP*, *ALB*, *TAD*, *TDO*, *MRP2*, *MDR*, *ASGPR1*, *CPS1*, and *G6PC*), genes related to phase I (CYP1A2, *CYP3A4*, *CYP3A7*, *CYP2B6*, *CYP2C9*), phase II (*UGT2B7* and *UGT2B15*), and phase III (*SICO2B1*, *OATPIB1*, and *AQP7*) of drug metabolism, cytoskeleton genes (*ITGα* and *ITGβ*) and downstream genes related to their signaling pathway (*FOXA2*, *HNF4α*, and *PXR*) in the LOs and BLOs and fetal and adult liver tissues as control groups ($n = 4$). Data were normalized against *GAPDH* and are presented as fold change compared with those of co-culture day 0 as the calibrator. Data are shown as mean ± SD ($n = 3$). Statistical analysis was performed using unpaired two-tailed Student's *t*-test. * $p < 0.05$ and ** $p < 0.01$. (LOs: liver organoids; BLOs: bioengineered liver organoids; HE: hepatic endoderm; iPSC4: induced pluripotent stem cell 4).

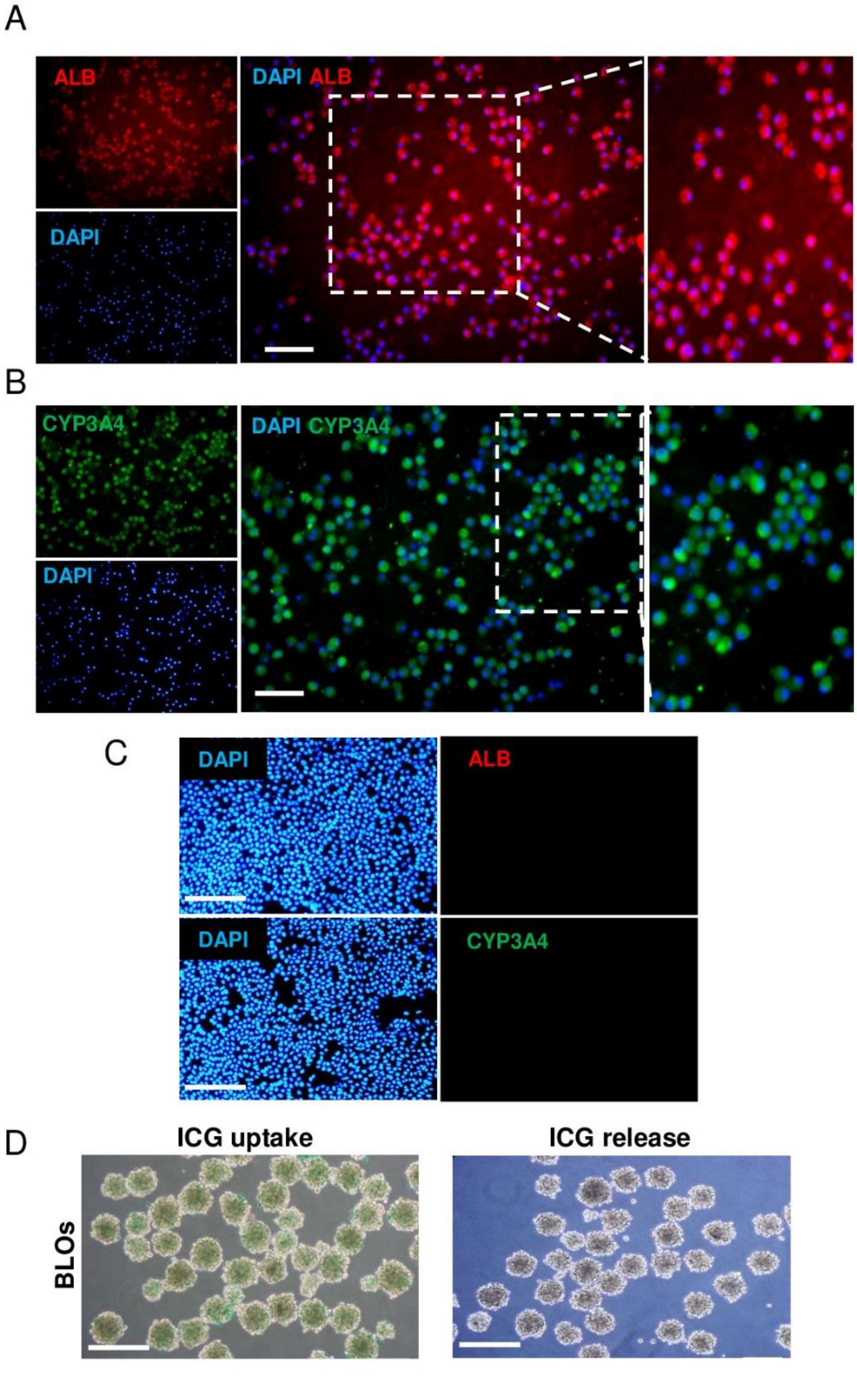

Figure S6. Immunostaining of primary human hepatocyte and fibroblast and ICG assay of BLOs. **(A, B)** Immunostaining of primary human hepatocyte using direct antibodies against ALB and CYP3A4 in 2D culture (Scale bare: 100 μm). **(C)** Immunostaining of fibroblast using direct antibodies against ALB and CYP3A4 in 2D culture. The nuclei were counter-stained with DAPI (scale bare: 200 μm). **(D)** ICG uptake and release in the BLOs group (Scale bare: 500 μm). (ALB: albumin; 2D: 2 dimensional; ICG; indocyanine green).

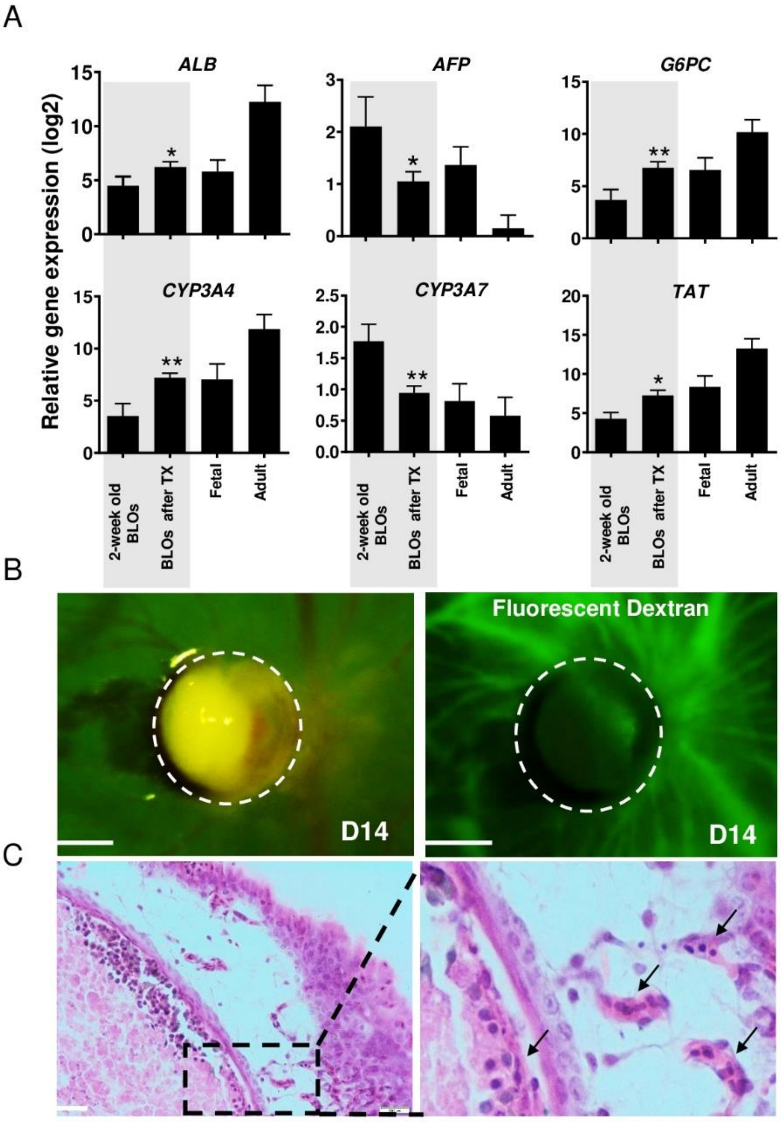

Figure S7. *Ex ovo* implantation of the BLOs. **(A)** Implantation of one-day-old BLOs on *ex ovo* chorioallantoic membrane. Fluorescence microscopy images taken before and after dextran injection (left) (scale bar 200 µm) and **(B)** H&E-stained section of BLOs 14 days after transplantation (right) (arrows referring to the capillaries are penetrating into the implanted BLOs, scale bar: 200 µm). **(C)** Relative gene expression of hepatic-specific genes (*ALB*, *AFP*, *G6PC*, *CYP3A4*, *CYP3A7*, and *TAD*) in two-week-old BLOs, before transplantation and two weeks after transplantation compared with the fetal and adult liver tissues as control groups. Data were normalized against *GAPDH* and are presented as fold change compared with data obtained on co-culture day 0 as the calibrator. Data are shown as mean±SD (n=3). Statistical analysis was performed by using unpaired two-tailed Student's *t*-test. * $p < 0.05$, and ** $p < 0.01$. (LOs: liver organoids; BLOs: bioengineered liver organoids).

Table S1. Optimization of cell/MP and cell/microwell ratios for successful cell aggregate formation.

Cell types and Ratio	Cell/MP ratio	Cell/Microwell	Condensation
HE:HUVEC:MSC (10:7:2)	1:1	1000	-
HE:HUVEC:MSC	3:1	1000	-

(10:7:2)			
HE:HUVEC:MSC		1000	-
(10:7:2)	2:1		-
HE:HUVEC:MSC (10:7:2)	1:1	500	-
HE:HUVEC:MSC	3:1	500	+
(10:7:2)	2:1		

Table S2. Human Primer sequences used in qRT-PCR.

Gene	Forward	Reverse
CXCR4	CAC CGC ATC TGG AGA ACC A	GCC CAT TTC CTC GGT GTA GTT
FOXA2	GGA GCG GTG AAG ATG GAA	TAC GTG TTC ATG CCG TTC AT
SOX17	CTC TGC CTC CTC CAC GAA	CAG AAT CCA GAC CTG CAC AA
HHEX	AAGATTTGCCCAGTGAACAG	TTTATCGCCCTCAATGTCC
AFP	AAA TGC GTT TCT CGT TGC TT	GCC ACA GGC CAA TAG TTT GT
ALBUMIN	CTT CCT GGG CAT GTT TTT GT	TGG CAT AGC ATT CAT GAG GA
AAT	ACCTGATCGAGAATATAGACC	CTCCAGAAAGAAATCTTCCTC
TTR	GAGGAGGAATTTGTAGAAGGGA	CGTGGTGGAATAGGAGTAGG
HNF4α	CGATGACAATGAGTATGCCT	GTCGTTGATGTAGTCCTCCA
TAT	ATG CTG ATC TCT GTT ATG GG	CAC ATC GTT CTC AAA TTC TGG
CYP3A4	TTTTTGGATCCATTCTTTCTCTCAA	ATCCACTCGGTGCTTTTGTG
CYP3A7	GACCGTAAGTGGAGCCTGATTTC	ACAGACCATGAGAGAGCACAA
TDO	GGT TTA GAG CCA CAT GGA TT	ACA GTT GAT CGC AGG TAG TG
G6PC	GTG GAT TCT CTT TGG ACA GC	AGC AGC AAG GTA GAT TCG TG
GAPDH	CTC ATT TCC TGG TAT GAC AAC GA	CTT CCT CTT GTG CTC TTG CT
UGT2B7	GATCCCAACAACTCATCCGCT	CAGCAGCTCACTACAGGGAA
UGT2B15	CCAACCAATGAAGCCCCTG	GTTGTGAGCTGCGACTCGAA
SLCO1B1	TTGGAGGTGTTTTGACTGCTT	ACAAGTGGATAAGGTCGATGTTG
AQP7	ACCCGTGGCTCCAAAATGG	GGAACCAAGGCCGAATACCA
SLCO2B1	GGCAAGGACTCTCCCTCTAAG	GTTTGGTGCAATCTGGACTAGG
CYP1A2	ATGCTCAGCCTCGTGAAGAAC	GTTAGGCAGGTAGCGAAGGAT
ITGa	GCCTGTGGAGTACAAGTCCTT	AATTCGGGTGAAGTTATCTGTGG
ITGb	CAAGAGAGCTGAAGACTATCCCA	TGAAGTCCGAAGTAATCCTCCT
PXR	GACATGTGAAGGATGCAAGG	CTCTCCAGGCACTTGCGCA
CYP2B6	GCACTCCTCACAGGACTCTTG	CCCAGGTGTACCGTGAAGAC
CYP2C9	GCCTGAAACCCATAGTGGTG	GGGGCTGCTCAAAATCTTGATG
MDR	CTCTATCTCTCCCGACATGACC	AGCAGACGATCCACAGCAAAA
TBX3	GAGGCTAAAGAACTTTGGGATCA	CATTTCGGGGTCGGCCTTA
MRP2	CCCTGCTGTTCGATATACCAATC	TCGAGAGAATCCAGAATAGGGAC
CPS1	AATGAGGTGGGCTTAAAGCAAG	AGTTCCACTCCACAGTTCAGA
GAPDH	CTCATTTCCTGGTATGACAACGA	CTTCCTCTTGTGCTCTTGCT

Table S3. List of antibodies used for immunoflourescent staining.

Primary antibody	Type	Company	Cat. No.	Dilution

**ALBUMIN (ALB)**	Goat	Bethyl Laboratories	A80-229A	1:100
**E-CADHERIN**	Mouse	Santa Cruz	SC8426	1:100
**CYP3A4**	Mouse	Santa Cruz	SC53850	1:50
**CYP1A1**	Mouse	Santa Cruz	SC-25304	1:50
**ZO-1**	Rabbit	Abcam	Ab59720	1:100
**CD-31**	Rabbit	Abcam	Ab28364	1:100
**VIMENTIN**	Mouse	Abcam	Ab128507	1:100
**Secondary antibody**	Type	Company	Cat. No.	Dilution
**Anti-goat IgG Alexa Fluor®568**	Donkey	Invitrogen	A10036	1:300
**Anti-mouse IgG Alexa Fluor®488**	Donkey	Invitrogen	A21202	1:500
**Anti-rabbit IgG Alexa Fluor®488**	Donkey	Invitrogen	A21206	1:500
**Anti-mouse IgG Alexa Fluor®546**	Donkey	Invitrogen	A10036	1:500
**Anti-rabbit IgG Alexa Fluor®546**	Donkey	Invitrogen	A10040	1:500

Table S4. Data for Log Rank Test to Compare Survival Curves.

**Time, days**	**Number at Risk - Group 1 (sham) N1t**	**Number at Risk - Group 2 (treated) N2t**	**Number of Events (death) - Group 1(sham) O1t**	**Number of Events (death) - Group 2 (treated) O2t**
**0**	8	8	0	0
**2**	6	8	2	0
**4**	5	8	1	0
**6**	4	8	1	0
**8**	4	8	0	0
**10**	4	8	0	0
**12**	4	8	0	0
**14**	4	8	0	0

We next total the number at risk, Nt = N1t+N2t, at each event time and the number of observed events (deaths), Ot = O1t+O2t, at each event time. We then compute the expected number of events in each group. The expected number of events is computed at each event time as follows:

E1t = N1t*(Ot/Nt) for group 1 and E2t = N2t*(Ot/Nt) for group 2. The calculations are shown in the table below.

Table S5. Expected Numbers of Events in Each Group.

**Time, days**	**Number at Risk in Group 1 (sham) N1t**	**Number at Risk in Group 2 (treated) N2t**	**Total Number at Risk Nt**	**Number of Events in Group 1 O1t**	**Number of Events in Group 2 O2t**	**Total Number of Events Ot**	**Expected Number of Events inGroup 1 (sham) E1t = N1t*(Ot/Nt)**	**Expected Number of Events in Group 2 (treated) E2t = N2t*(Ot/Nt)**
**0**	8	8	16	0	0	0	0	0
**2**	6	8	14	2	0	2	0.85	1.14

**4**	5	8	13	1	0	1	0.38	0.61
**6**	4	8	12	1	0	1	0.33	0.66
**8**	4	8	12	0	0	0	0.33	0.66
**10**	4	8	12	0	0	0	0.33	0.66
**12**	4	8	12	0	0	0	0.33	0.66
**14**	4	8	12	0	0	0	0.33	0.66

we next sum the observed numbers of events in each group (∑O1t and ΣO2t) and the expected numbers of events in each group (ΣE1t and ΣE2t) over time. These are shown in the bottom row of the next table below.

Scheme 6. Total Observed and Expected Numbers of Observed in each Group.

Time, days	Number at Risk in Group 1 (sham) N1t	Number at Risk in Group 2 (treated) N2t	Total Number at Risk Nt	Number of Events in Group 1 O1t	Number of Events in Group 2 O2t	Total Number of Events Ot	Expected Number of Events in Group 1 (sham) E1t = N1t*(Ot/Nt)	Expected Number of Events in Group 2 (treated) E2t = N2t*(Ot/Nt)
**0**	8	8	16	0	0	0	0	0
**2**	6	8	14	2	0	2	0.85	1.14
**4**	5	8	13	1	0	1	0.38	0.61
**6**	4	8	12	1	0	1	0.33	0.66
**8**	4	8	12	0	0	0	0.33	0.66
**10**	4	8	12	0	0	0	0.33	0.66
**12**	4	8	12	0	0	0	0.33	0.66
**14**	4	8	12	0	0	0	0.33	0.66
							2.88	5.05

We can now compute the test statistic:

$$\chi^2 = \Sigma \frac{(\Sigma O_{jt} - \Sigma E_{jt})^2}{\Sigma E_{jt}} \quad (1)$$

For this test, the decision rule is to Reject H0 if X2 > 3.84. We observe X2 = 5.48, which exceeds the critical value of 3.84. Therefore, we reject H0. We have significant evidence, α=0.05, to show that the two survival curves are different.
